# Effect of Thermal Treatment on Gelling and Emulsifying Properties of Soy β-Conglycinin and Glycinin

**DOI:** 10.3390/foods13121804

**Published:** 2024-06-08

**Authors:** Wei Zhang, Mengru Jin, Hong Wang, Siqi Cheng, Jialu Cao, Dingrong Kang, Jingnan Zhang, Wei Zhou, Longteng Zhang, Rugang Zhu, Donghong Liu, Guanchen Liu

**Affiliations:** 1 Center for Sustainable Protein, DeePro Technology (Beijing), Beijing 101200, China; w.zhang@thexmeats.com (W.Z.); a.cheng@thexmeats.com (S.C.);; 2 Center for Alternative Protein, Beijing 101200, China; 3 Light Industry College, Liaoning University, Shenyang 110036, China; 4 School of Food Science and Engineering, Hainan University, Haikou 570228, China; 5 Innovation Center of Yangtze River Delta, Zhejiang University, Jiaxing 314100, China; 6 College of Biosystems Engineering and Food Science, Zhejiang University, Hangzhou 310058, China

**Keywords:** soy protein ingredients, thermal treatment, emulsification, gelation properties, 11S/7S ratio

## Abstract

This study investigated the impact of different preheat treatments on the emulsifying and gel textural properties of soy protein with varying 11S/7S ratios. A mixture of 7S and 11S globulins, obtained from defatted soybean meal, was prepared at different ratios. The mixed proteins were subjected to preheating (75 °C, 85 °C, and 95 °C for 5 min) or non-preheating, followed by spray drying or non-spray drying. The solubility of protein mixtures rich in the 7S fraction tended to decrease significantly after heating at 85 °C, while protein mixtures rich in the 11S fraction showed a significant decrease after heating at 95 °C. Surprisingly, the emulsion stability index (ESI) of protein mixtures rich in the 7S fraction significantly improved twofold during processing at 75 °C. This study revealed a negative correlation between the emulsifying ability of soy protein and the 11S/7S ratio. For protein mixtures rich in either the 7S or the 11S fractions, gelling proprieties as well as emulsion activity index (EAI) and ESI showed no significant changes after spray drying; however, surface hydrophobicity was significantly enhanced following heating at 85 °C post-spray drying treatment. These findings provide insights into the alterations in gelling and emulsifying properties during various heating processes, offering great potential for producing soy protein ingredients with enhanced emulsifying ability and gelling property. They also contribute to establishing a theoretical basis for the standardized production of soy protein isolate with specific functional characteristics.

## 1. Introduction

With the continuous growth of the population, animal-based protein sources will be insufficient to meet the increasing demand for protein food [1]. Simultaneously, traditional animal husbandry faces challenges in protein supply due to limitations in land, water, and greenhouse gas emissions [2]. Consequently, exploring and utilizing plant-derived proteins has emerged as a new solution for future protein supply. Soybean, as one of the most significant economic crops globally with extensive cultivation and high application value, offers soy protein that exhibits remarkable potential in enhancing various processing properties essential for food production. These properties include emulsification, gelation, foaming, and water and fat absorption—all widely recognized attributes within the food industry [3]. The functional properties of soybean protein are influenced by factors such as variety, origin, growing conditions, and processing methods; thus, effective control over these properties is crucial for industrial applications [4]. In-depth research on the relationship between protein components’ structure and functional properties in soybeans is highly significant, along with investigating changes in physical–chemical characteristics during heating processes. Such research ensures product consistency while driving advancements in future plant-based food development.

In soybean protein, the main components are 11S globulin (glycinin) and 7S globulin (β-conglycinin), with 11S accounting for approximately 30% and 7S accounting for about 40% [4]. Soy glycinin is a hexamer composed of five major subunits, each consisting of acidic and basic peptides linked by a disulfide bond, except for the acidic polypeptide [5]. β-conglycinin is a trimeric glycoprotein comprising three glycosylated subunits (α′, α, and β). These subunits primarily associate through hydrophobic interactions and hydrogen bonding. However, α′ and α subunits contain some cysteine content that can form small amounts of high-molecular-weight aggregates via disulfide linkage [6]. Due to inherent structural differences between the 7S and 11S globulins, they exhibit distinct physicochemical functions in soy protein. Fukushima [7] reported significant variations in the physicochemical properties of soy proteins among different cultivars due to varying proportions of 7S and 11S. It has been documented that while the 7S globulin demonstrates greater emulsifying activity, gelling property in soy protein ingredients is predominantly influenced by the presence of 11S [8]. Damodaran and Kinsella [9] observed that the dissociation of the 11S subunits is inhibited by the presence of 7S. Electrostatic interactions between the B subunit of 11S and β subunit of 7S also contribute to macromolecule aggregation which significantly affects gel properties [10]. Commercial sources provide two main types of soybean protein ingredients based on their protein content: soy protein isolates (SPIs) and soy protein concentrates (SPCs). These differ significantly in terms of functional properties [11]. There are numerous investigations into the gelling and emulsifying properties of soy protein. However, limited research has focused on individual soy β-conglycinin and glycinin, as well as their blends, both before and after spray drying.

Heating is a widely employed technique for enhancing the functional properties of soy protein, leading to diverse advantageous structural modifications [12]. However, when combined with the modification in the 11S/7S ratio, heating can induce numerous desirable changes in both structure and function. This study aims to evaluate the impact of different ratios of 11S/7S and heat treatments ranging from 75 °C to 95 °C on the gelation and emulsifying properties of soy protein isolate (SPI). The varying proportions of 7S and 11S globulin in different soybean varieties result in the distinct functional characteristics of soybean protein isolates. This work contributes significantly to the understanding of the gelling and emulsifying properties of plant protein, particularly soy β-conglycinin and glycinin, during heat processing. Understanding this mechanism could provide novel insights into achieving standardized production of soy protein isolates with specific gel properties and emulsifiability, crucial for tailoring the desirable functional properties of plant-derived proteins to meet future protein supply needs.

## 2. Materials and Methods

### 2.1. Materials and Chemicals

The low-temperature defatted soybean meal was obtained from Wonderful Biotechnology Co., Ltd. (Dongying, China). The reagents and chemicals utilized in the experiments were procured from Sinopharm Chemical Reagent Co., Ltd. (Shanghai, China), Macklin Chemical Co., Ltd. (Shanghai, China), Yihai Kerry Arawana Holdings Co., Ltd. (Shanghai, China), and Jinyuanxingke Technology Co., Ltd. (Beijing, China).

### 2.2. Preparation of Soy 7S Globulin and 11S Globulin

The 7S globulin and 11S globulin were prepared according to the extraction methods described by Nagano et al. [13], with slight modifications. The defatted soybean meal was pulverized and then passed through a 60-mesh sieve. The sieved soybean meal was dispersed into water (1:10, *w*/*v*) and adjusted to pH 8 using 2 M NaOH. The dispersion was stirred for 1 h at 40 °C, followed by centrifugation for 30 min at 7000 rpm at 4 °C. The sediment was dispersed in water (1:5, *w*/*v*) and the above steps were repeated once more. The supernatants were adjusted to pH 6.4 with 2 M HCl and centrifuged (6500 rpm, 20 min, 4 °C) to obtain the sediment. The sediment was dissolved in water and adjusted to pH 7.0, resulting in the isolation of the 11S globulin after freeze drying. The supernatants were further adjusted to pH 5 and NaCl was added to a concentration of 0.25 M, and the insoluble material (intermediate fraction) was removed by centrifugation at 7000 rpm for 30 min. After adding doubled ice water, the obtained supernatant was further adjusted to pH 4.8 and centrifuged (6500 rpm, 20 min, 4 °C) for the sediment. The sediment was dissolved in water and adjusted to a pH of 7.0, followed by the acquisition of 7S globulin through freeze drying.

### 2.3. Preparation of Protein Samples

The extracted 11S globulin and 7S globulin were combined in specific proportions. Based on the content of β-conglycinin (7S globulin) and glycinin (11S globulin), as well as the other proteins present in the isolated protein fractions, the ratio of glycinin to total protein was determined. Protein mixtures containing 30%, 45%, and 90% of glycinin relative to total protein (with the corresponding percentages of β-conglycinin being 61%, 47%, and 7%) were obtained, referred to as 11S-30 (representing β-conglycinin), 11S-45 (representing a soy protein isolate consisting of both β-conglycinin and glycinin), and 11S-90 (representing glycinin). These mixtures were dissolved in deionized water at a concentration of 10% (*w*/*v*). After complete hydration, the pH was adjusted to 7.0 for subsequent treatments. Each solution was divided into 6 portions for different thermal treatments. Portions 1–4 underwent non-thermal treatment or treatment at 75 °C, 85 °C, or 95 °C for 5 min followed by freeze drying. Portions 5–6 underwent non-thermal treatment followed by heating at 85 °C for 5 min and then spray drying (inlet air temperature: 180 °C; outlet air temperature: 80 °C). The commercial SPI served as a control.

### 2.4. Protein Dispersibility Index (PDI)

The protein dispersibility (PDI) was determined on a 0.5 g sample following the method described by Iwe et al. [14]. The sample was blended with 12.5 mL of distilled water for 10 min using a Waring Blender operated at 8500 rpm and room temperature for 15 min. After allowing the slurry to settle for 10 min, the decantate was separated and centrifuged at 2800 rpm (610 g) for 10 min. Subsequently, 5 mL of the supernatant was transferred into a Kjeldahl tube for further treatment. The protein dispersion index (PDI) can be calculated using the following formula:

PDI (%) = C′/C × 100%(1)

where C′ represents the protein content in the supernatant and C represents the total protein content in the weighed soybean protein sample.

### 2.5. Surface Hydrophobicity

The surface hydrophobicity was determined using the method described by Chelh et al. [15]. To 1 mL of protein solution (10 mg/mL protein, pH 7.0), 200 μL of 1 mg/mL BPB (in distilled water) was added and thoroughly mixed. A control group, without myofibrils, consisted of adding 200 μL of 1 mg/mL BPB to 10 mL of a 10 mM phosphate buffer at pH 7. Both samples and control were agitated at room temperature for 20 min and then centrifuged for 10 min at a speed of 5000 g. The absorbance of the diluted supernatant (diluted at a ratio of 1/10) was measured at a wavelength of 595 nm against a blank consisting only of phosphate buffer. The formula used was as follows:

A (μg) = 200 × ((A_1_ − A_2_)/A_1_ × 100%) (2)

where A represents the surface hydrophobicity, A_1_ represents the absorbance value in the blank group, and A_2_ represents the absorbance value in the sample.

### 2.6. Viscosity

The viscosity measurements were conducted using a rotary viscometer equipped with a No.05 rotor. Samples were dispersed into distilled water (1:10, *w*/*v*), and the temperature of the protein solution was controlled using a constant-temperature bath. The solution was initially set at 25 °C for 5 min, then heated to 85 °C for 15 min, and finally cooled back to 25 °C for another 5 min. The rotational speed was fixed at 160 rpm, and data were recorded every 10 s.

### 2.7. Emulsifying Properties

Emulsions of mixed protein samples were prepared by combining 5 mL of sunflower seed oil with 15 mL of protein solutions (10 mg/mL, pH 7) using a high-speed homogenizer (THR-300-28, Jingqi Instrument, Shanghai, CN) at 15,000 rpm for 2 min. The emulsifying activity index (EAI) and emulsion stability index (ESI) were determined following the method described by Pearce &Kinsella [16]. In brief, 50 μL of fresh emulsion was added into 5 mL 0.1% SDS solution and mixed well. The absorbance of the diluted emulsion was then measured at 500 nm using a UV–Visible spectrophotometer (721N, Yidian Science Devices, Shanghai, China). After an incubation period of 10 min., another sample consisting of 50 μL emulsion was taken and measured using the same procedure. A reference measurement was performed using a solution containing only the diluent agent (0.1% SDS). The EAI value was calculated according to the following equation:

EAI (m^2^/g) = (2 × 2.303 × A_0_ × DF)/(c × θ × 10,000) (3)

where A_0_ represents the initial absorbance value at 0 min in 500 nm; DF is the dilution factor; c denotes the protein concentration in g/mL; and θ indicates the volume fraction of sunflower seed oil.

The ESI value was determined as follows:

ESI (min) = A_0_ × ∆t/∆A (4)

Herein, A_0_ refers to the initial absorbance value at 0 min in 500 nm; Δt represents the incubation time duration in minutes; and ΔA signifies the difference between absorbance values at 0 min and after incubation.

### 2.8. Gel Strength

The SPI solution (12%, *w*/*v*) was injected into a 20 mL disposable syringe. The lower end of the syringe was sealed with a rubber stopper, and the upper end was covered with a film. Subsequently, the syringe was immersed in a water bath at 95 °C for 40 min and rapidly cooled in an ice water bath for 15 min. After overnight refrigeration at 4 °C, the SPI gel was obtained.

The gel strength analysis was conducted using TA-XI plus texture analyzer (Stable Microsystems, Godalming, UK), following the method described by Peng and Guo [17]. Cylindrical gel samples measuring 20 mm in diameter and 10 mm in height were prepared. The probe (TA/0.5, 12.7 mm) penetrated the gels at a constant speed of 1 mm/s until reaching a trigger force of 5 g with deformation limited to 30%. Hardness (gram-force, gf) was calculated to evaluate the gel strength of the protein mixtures.

### 2.9. Statistical Analysis

Statistical analyses were performed using Statistix v9.0 software (Analytical Software, Tallahassee, FL, USA). All experiments were conducted in triplicate, and the data presented in the figures and tables are expressed as mean ± standard deviation. The significance level was set at *p* < 0.05 based on two-way ANOVA.

## 3. Results

### 3.1. Solubility

**Figure 1:**
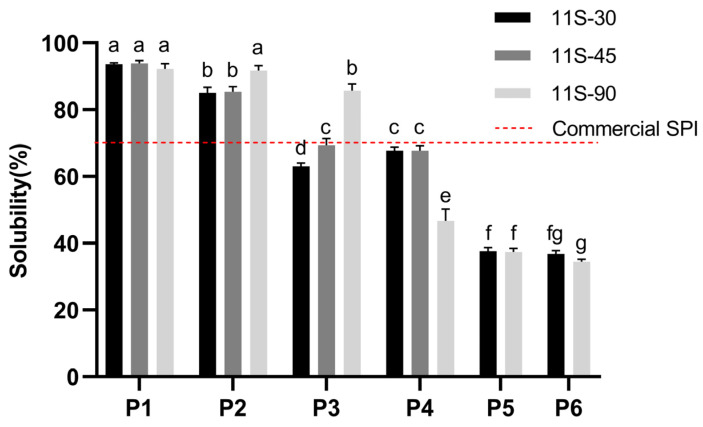
Solubility of protein mixtures with three different 11S/7S ratios treated at different preheating temperatures and with different drying methods (P1-4: portions subjected to non-thermal treatment, or treatment at 75 °C, 85 °C and 95 °C for 5 min then freeze-dried; P5-6: portions subjected to non-thermal treatment or treatment at 85 °C for 5 min then spray-dried). Values are presented as mean values and error bars represent the standard deviations of three independent experiments. Different lowercase letters (a–g) indicate significant differences among treatments (*p* < 0.05).

Solubility is a crucial functional property of proteins and is closely associated with their other functional properties [18]. The solubility characteristics of protein ingredients depend on various processing parameters, including solvent type, pH, ionic strength, mechanical forces, and temperature [19]. As depicted in Figure 1, there were no significant differences observed among the different protein proportions. The results demonstrated that both the 7S and 11S globulins exhibited excellent dispersion ability in aqueous solution systems at varying proportions. However, the protein dispersion coefficients of 11S-30 and 11S-45 significantly decreased after heating at 75 °C. For 11S-90, there was no significant difference observed after heating at temperatures of 75 °C or 85 °C; however, a notable decrease was observed after heating at 95 °C. This decline in solubility can be attributed to protein denaturation, since the denaturation temperatures for the respective globulins are approximately around 75 °C and 90 °C [20,21]. Due to the denaturation of the 7S globulin above its critical temperature (75 °C), more hydrophobic groups became exposed, leading to aggregation and subsequently decreasing its dispersion ability [22]. Moreover, as the 11S/7S ratio increased within the protein mixture, the denaturation temperature also gradually rose, resulting in higher temperatures being required to affect their structural integrity [23]. Notably, intense heating had a pronounced impact on solubility for samples with high 11S/7S ratios but had less apparent effects on those with low 11S/7S ratios. Guo et al. [24] reported that the addition of the 7S globulin terminated the assembly between the 11S aggregates, potentially attributed to the interaction between the 7S globulin and the highly insoluble basic peptide of 11S globulin, thereby enhancing its solubility. These findings were further corroborated by surface hydrophobicity analysis.

The dispersion coefficients of the 11S-30 and 11S-90 proteins, with and without spray drying, were determined. The solubility of the unheated 11S-30 and 11S-90 proteins exhibited a significant decrease after spray drying. However, no significant difference was observed between the solubility of the 7S and 11S proteins following heat treatment at 85 °C for 5 min (P5 vs. P6 in Figure 1). These findings suggest that the structural integrity of both proteins may have been irreversibly compromised due to excessive temperature during the spray drying process.

### 3.2. Surface Hydrophobicity

**Figure 2:**
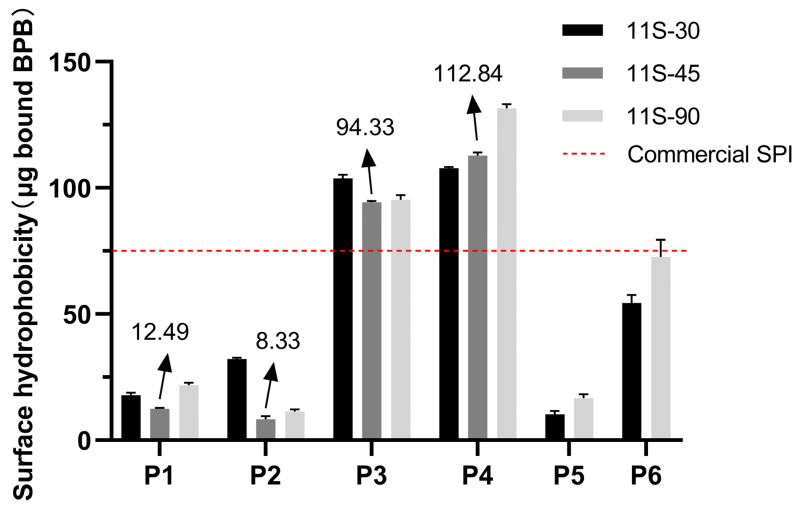
Surface hydrophobicity of protein mixtures with three different 11S/7S ratios treated at different preheating temperatures and with different drying methods (P1-4: portions subjected to non-thermal treatment, and treated at 75 °C, 85 °C, or 95 °C for 5 min then freeze-dried; P5-6: portions subjected to non-thermal treatment or treatment at 85 °C for 5 min then spray-dried). Values are presented as mean values and error bars represent the standard deviations of three independent experiments.

As depicted in Figure 2, the surface hydrophobicity of proteins with varying 11S/7S ratios was observed to be below 25 µg in the P1 group, and did not bind more bromophenol blue. These results suggest that alkali extraction and acid precipitation had minimal impact on the conformation of the 7S and 11S globulins, as well as the limited exposure of hydrophobic groups for bromophenol blue binding. During heat treatment, protein aggregation involves intricate intermolecular interactions, primarily driven by hydrophobic associations [25]. Elevated temperatures affected protein conformation, leading to the increased exposure of hydrophobic groups and binding sites, thereby promoting intermolecular hydrophobic association [26]. Figure 2 demonstrates a substantial increase in surface hydrophobicity for protein mixtures with different 11S/7S ratios from 75 °C to 85 °C due to heat-induced unfolding exposing the inner hydrophobic groups [27]. In the P2 group, a slight decrease in surface hydrophobicity was observed with an increasing 11S/7S ratio due to the easier modification of globulin through heat treatment attributed to the higher denaturation temperature for 11 S and lower denaturation temperature for 7S.

The surface hydrophobicity of 11S-30 without heat treatment showed no significant difference before and after spray drying (P1 group vs. P5 group). However, the preheating treatment at 85 °C for 5 min significantly enhanced the surface hydrophobicity after spray drying (P6 group vs. P1 group), even surpassing the initial level before spray drying (P6 group vs. P3 group). These findings indicate that thermal treatment effectively enhances the surface hydrophobicity.

### 3.3. Viscosity

**Figure 3:**
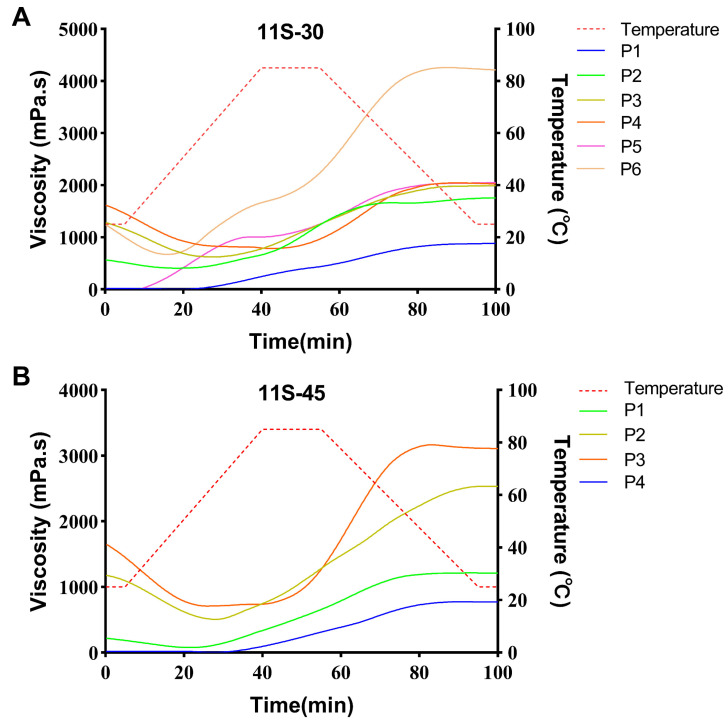

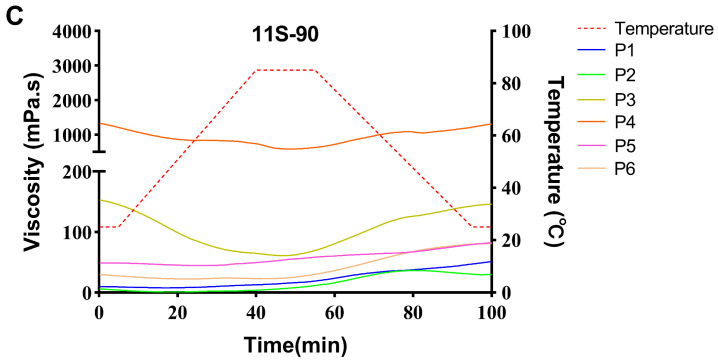
Viscosity of protein mixtures with three different 11S/7S ratios ((**A**) 11S-30; (**B**) 11S-45; (**C**) 11S-90) treated at different preheating temperatures and different drying methods (P1-4: portions subjected to non-thermal treatment, or treated at 75 °C, 85 °C, or 95 °C for 5 min then freeze-dried; P5-6: portions subjected to non-thermal treatment or treated at 85 °C for 5 min then spray-dried).

The variation in protein viscosity is closely associated with protein solubility, which can reflect the kinetics of protein–water interaction within the system [28]. As depicted in Figure 3, the viscosity levels of the three ratios of proteins were all below 50 mPa·s at the initial temperature of 25 °C. With increasing temperature, the viscosities of proteins at different proportions exhibited a significant increase, indicating conformational changes primarily attributed to thermal denaturation temperatures [25]. Upon heating the samples to 85 °C for 15 min and subsequent cooling to 25 °C, the viscosities of proteins at various proportions continued to rise. However, after the completion of cooling (100 min), it was observed that protein viscosity displayed an inverse relationship with the 11S/7S ratio. The viscosity reached as high as 910 mPa·s for 11S-30 while only reaching a non-significantly different value of 52 mPa·s for 11S-90 compared to its initial viscosity. Furthermore, an increase in the denaturation temperature of proteins was observed with higher values of the 11S/7S ratio due to the predominant role played by hydrophilic groups resulting in lower viscosity [20].

The viscosity results of the protein mixtures after heat treatments are presented in Figure 3. The initial viscosity of 11S-30 increases with the rise in preheating temperature. Similar observations were made for the other two protein proportions. As depicted in Figure 3A, the viscosity of 11S-30 tends to stabilize after different heating conditions at 100 min, albeit at a higher value than the initial viscosity. The range of viscosity change decreases as the protein preheating temperature increases, indicating that lower heating temperatures (75 °C) result in less modification of the 7S protein fraction. When it is heated again at 85 °C for 15 min, further denaturation of globulin leads to higher viscosity. Conversely, higher temperatures (85 °C and 95 °C) cause more extensive protein denaturation. Upon subjecting it to heat treatment again at 85 °C for an additional duration of 15 min, no further changes occurred in both protein structure and viscosity levels. The viscosity of proteins preheated at 75 °C and 85 °C for 11S-90 showed no significant difference. However, a much higher viscosity was observed after preheating at 95 °C, while there was no significant difference in viscosity from the beginning to the end when heated at 85 °C for 15 min. Consequently, high heat treatment resulted in the improved thermal stability of 11S-90, whereas heat treatments at 75 °C and 85 °C had negligible effects on its properties [29].

The initial viscosity of the non-heated 11S-30 protein before and after spray drying was observed to be low with no significant difference. Similarly, the preheated 11S-30 protein exhibited higher initial viscosity before and after spray drying, but still without any significant difference. However, upon further heating at 85 °C for 15 min, a notable disparity in viscosity was observed between the preheated 11S-30 protein before and after spray drying. Specifically, the viscosity of the protein prior to drying reached 1990 mPa·s, whereas the spray-dried protein displayed a significantly increased viscosity of up to 4192 mPa·s. This observation suggests that preheating the 11S-30 protein results in reduced heat stability post-spray drying, leading to the exposure of more hydrophobic groups. On the other hand, there were no significant differences in either the initial and final viscosities among all samples of the 11S-90 proteins tested; this can be attributed to the higher denaturation temperatures of the 11S glycinin [20].

### 3.4. Emulsifying Properties

**Figure 4:**
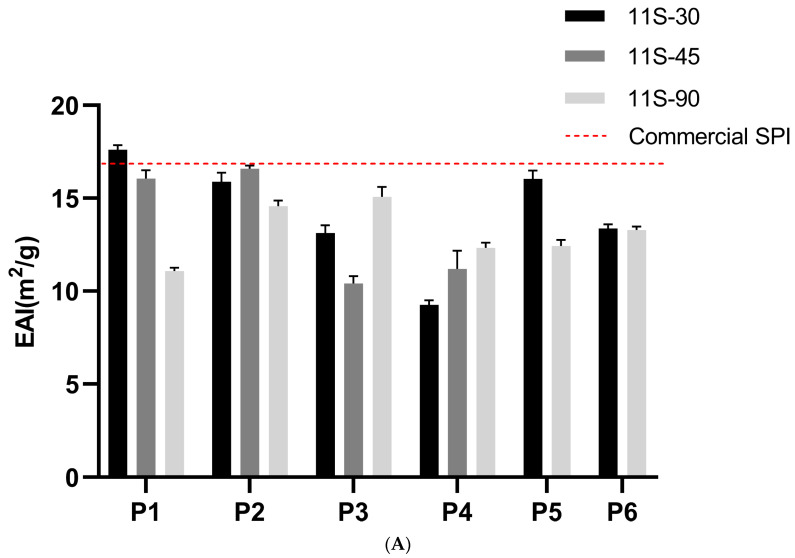

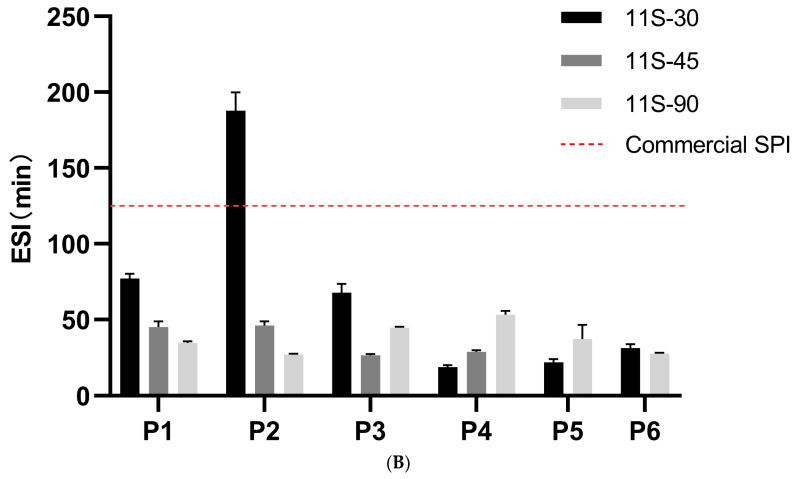
EAI (**A**) and ESI (**B**) of protein mixtures with three different 11S/7S ratios treated at different preheating temperatures and with different drying methods (P1-4: portions subjected to non-thermal treatment, or treated at 75 °C, 85 °C, or 95 °C for 5 min then freeze-dried; P5-6: portions subjected to non-thermal treatment or treated at 85 °C for 5 min then spray-dried). Values are presented as mean values and error bars represent the standard deviations of three independent experiments.

The higher level of 7S in the P1 and P5 groups was found to be associated with increased emulsifying properties (Figure 4). This can be attributed to the significant role played by carbohydrate moieties in 7S globulin, which enhance its emulsifying property [30]. The cohesion of 7S globulin molecules, both intra- and intermolecularly, results in the formation of more ordered films, thereby improving emulsion stability [31]. Conversely, due to its low surface hydrophobicity, large molecular size, and limited flexibility, 11S exhibits slow migration and poor emulsification properties [32].

Heat treatments applied to soy proteins have a profound impact on their surface hydrophobicity/hydrophilicity balance as well as their aggregated state, consequently influencing their emulsibility and emulsion stability [33]. The EAI and ESI values for 11S-30 were negatively correlated with heating temperature; thus, thermal treatment did not enhance the emulsifying property of globulin. Compared with the unheated protein samples, heating at 95 °C resulted in decreases of 45% and 35% in EAI for 11S-30 and 11S-45, respectively. The trend in ESI with different ratios of 11S/7S, achieved through preheating at various temperatures, aligns consistently with the trend observed for EAI. Notably, preheating exerted a more pronounced influence on proteins exhibiting lower 11S/7S ratios. The EAI and ESI values for 11S-90 remained unaffected by temperature variations.

There was no significant difference in the EAI of the non-preheated 11S-90 globulin before and after spray drying. However, the EAI of the heated 11S-90 after spray drying was found to be 15% lower compared to before spray drying. These results indicate that spray drying improves the EAI of heated 11S globulin by further cross-linking the protein through the originally exposed sulfhydryl and hydrophobic groups post-heat treatment, resulting in larger protein particles and reduced emulsifying activity. Conversely, there was no significant difference in the EAI between the non-preheated and heated 11S-30 with or without spray drying due to the lower content of disulfide bonds in 7S compared to that in 11S.

### 3.5. Gel Strength

Heat treatment can induce the separation, denaturation, and aggregation of the 7S and 11S subunits, thereby resulting in protein gelation [4]. The gel strength of 11S-30 was significantly lower compared to that of 11S-90, indicating an increased involvement of hydrophobic groups and disulfide bonds in protein–protein cross-linking with a higher 11S/7S ratio. Wu et al. [34] reported a higher abundance of hydrophobic amino acids in the primary structure of basic polypeptides within the 11S protein subunit than any other subunits, leading to the formation of larger protein aggregates with higher hydrodynamic radius at an elevated 11S ratio.

After heat treatment, the gel strength of 11S-30 was significantly increased by threefold when heated at 75 °C for 5 min (as shown in the P2 group). However, no further improvement in gel strength was observed with additional thermal treatment (as shown in P3 and P4). For 11S-90, heat treatment slightly increased the gel strength starting from 85 °C but tended to decrease with higher thermal treatment (P3 vs. P4).

**Figure 5:**
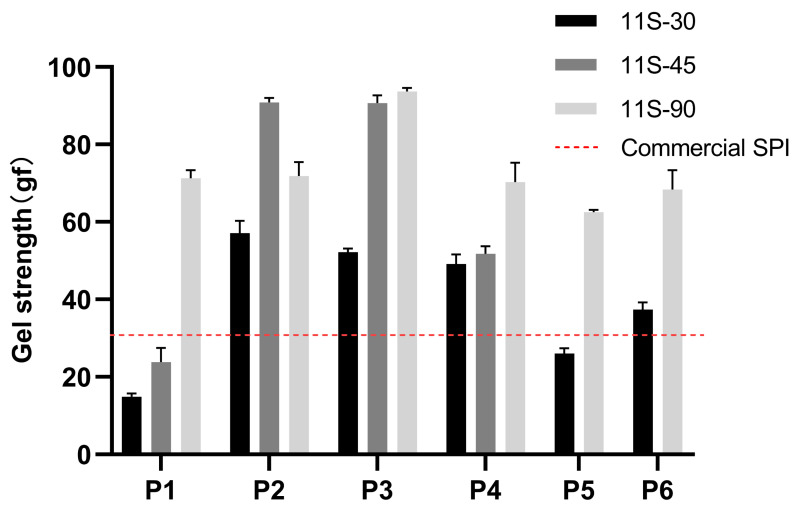
Gel strength of protein mixtures with three different 11S/7S ratios treated at different preheating temperatures and with different drying methods (P1-4: portions subjected to non-thermal treatment, or treated at 75 °C, 85 °C, or 95 °C for 5 min then freeze-dried; P5-6: portions subjected to non-thermal treatment or treated at 85 °C for 5 min then spray-dried). Values are presented as mean values and error bars represent the standard deviations of three independent experiments.

The gel strength of the non-heated 11S-30 after spray drying was significantly higher than before spray drying, as demonstrated in Figure 5. Conversely, the gel strength of the heated 11S-30 decreased significantly after spray drying. This suggests that native proteins with a low 11S/7S ratio exhibit lower denaturation compared to heated proteins, resulting in an increase in gel strength. Moreover, it indicates that the high temperature during spray drying leads to a greater degree of protein aggregation for those with a high 11S/7S ratio, thereby preventing the formation of a dense protein network. Additionally, heat treatments at 85 °C for both 7S and 11S contributed slightly to gelling strength after spray drying (P5 vs. P6).

## 4. Conclusions

The functional properties of soy protein ingredients were strongly and directly related to the 11S/7S ratio and the degree of heat treatments. These results indicate that protein mixtures rich in the 7S fraction exhibit a superior emulsifying ability compared to those enriched in the 11S fraction. The solubility, ESI, and EAI remained relatively stable throughout varying degrees of pre-spray drying heating treatment, suggesting that heat treatment is not an effective method for enhancing their functionality. However, significant changes in gelling property, surface hydrophobicity, and viscosity were observed with the increasing strength of heat treatment prior to spray drying, although some improvements reverted after spray drying. These findings underscore dynamic changes in gelling and emulsifying properties during various heating processes while suggesting great potential for producing soy protein ingredients with improved emulsification ability and gelling properties. Further investigation is needed to explore a wider range of heat treatments.

## Data Availability

The original contributions presented in the study are included in the article, further inquiries can be directed to the corresponding authors.
